# Supplementary material for: Determination of a Tumor-Promoting Microenvironment in Recurrent Medulloblastoma: A Multi-Omics Study of Cerebrospinal Fluid
Source: Cancers (Basel). 2020 May 26;12(6):1350. doi: 10.3390/cancers12061350 (PMC7352284; doi:10.3390/cancers12061350)
Supplement: Supplementary file 1 [file cancers-12-01350-s001.zip › Supplementary Information - Materials and Methods.pdf]

cancers

MDPI

Article

Determination of a Tumor-Promoting Microenvironment in Recurrent Medulloblastoma: A Multi-Omics Study of Cerebrospinal Fluid

Bernd Reichl, Laura Niederstaetter, Thomas Boegl, Benjamin Neuditschko, Andrea Bileck, Johannes Gojo, Wolfgang Buchberger, Andreas Peyrl and Christopher Gerner

Supplementary Information – Materials and Methods

Detailed description regarding sample preparation and instrumental analysis separately for each class of molecule, i.e. lipids, oxylipins, proteins and amino acids

Lipids

100 µL CSF were diluted in 400 µL methanol (VWR) containing two internal standards (LPC 17:0 and PC 17:0/17:0, Sigma-Aldrich) and vigorously mixed for 15 min at 10 °C on a thermoshaker. Samples were then centrifuged at 4000 g for 8 min at 10 °C to remove precipitated proteins. In parallel, a quality control sample (QC) was prepared by pooling aliquots from all individual clinical samples, followed by the same sample preparation method as applied for the individual samples. The collected supernatants were immediately analyzed employing a 1260 series high performance liquid chromatography system (Agilent Technologies) hyphenated with a LTQ Orbitrap XL mass spectrometer (Thermo Fisher Scientific). Samples were kept in a thermostated autosampler at 8 °C during the sequence. Chromatographic separation was achieved using a Kinetex C18 reversed phase column in the dimensions 150 × 3 mm and particle size of 2.6 µm (Phenomenex). Two solvent mixtures, A and B, were used as mobile phases for chromatographic separation. Solvent A was a 60:40 mixture of water (high purity water produced in house by Milli-Q water purification system, Millipore) and acetonitrile (HPLC gradient grade, VWR), containing 10 mM ammonium acetate (Merck) and 1 mM acetic acid (Merck). Solvent B was a 90:10 mixture of isopropyl alcohol (LC-MS grade, VWR) and acetonitrile. Starting conditions were set to 40% solvent A and 60% solvent B, which was kept constant for 8 min. Solvent B was then increased to 97% over a period of 17 min and kept constant again for 15 min. Post run time was set to 15 min for sufficient reequilibration of the column, resulting in a total run time of 55 min. The flow rate was set to 0.3 mL min^{-1}, the column was thermostated at 50 °C, and an injection volume of 7 µL was used. The mass spectrometer comprised an electrospray ionization (ESI) source which was run in the positive ionization mode. Sheath gas flow rate was set at 45 arbitrary units, spray voltage at 3500 V, capillary temperature at 350 °C, capillary voltage at 80 V and the tube lens at 150 V. The instrument was operated in a full scan acquisition mode with a mass resolution of 60,000 at m/z 400. The applied methodology allowed us to quantitatively assess 59 lipids representing six lipid classes, phosphatidylcholines (PC, including diacyl- and alkyl-acyl PC species), phosphatidylethanolamines (PE), lysophosphatidylcholines (LPC), lysophosphatidylethanolamines (LPE), sphingomyelins (SM) and ceramides (Cer). Structural confirmation of the target analytes was obtained in preliminary MS2 experiments. All lipids were analyzed in their protonated form. Raw files were analyzed by peak integration using Xcalibur Quan Browser (version 4.1.31.9; Thermo Fisher Scientific), followed by normalization against the internal standard (see complete lipid target list in Supplementary Table S1).

Oxylipins

200 µL of sample were diluted in 1 mL absolute ethanol (AustroAlco) containing 10-100 nM of each internal standard (12S-,15S-HETE-d8, 5-Oxo-ETE-d7, 11.12-DiHETrE-d11, PGE-d4, 20-HETE-d6;

Cayman Chemical) in 15 mL tubes (Falcon) and stored over night at -20 °C. Samples were then centrifuged for 30 min at 4536 g and 4 °C, and the supernatants were transferred into new 15 mL tubes. Ethanol was evaporated via vacuum centrifugation at 38 °C until the original sample volume was restored. Samples were then loaded on preconditioned 30 mg/mL StrataX solid phase extraction (SPE) columns (Phenomenex). Columns were washed with 2 mL MS grade water (VWR) and oxylipins were eluted with 500 µL methanol (MeOH; Merck) containing 2% formic acid (FA; Sigma-Aldrich). The eluates were subsequently stored in 2 mL glass vials at -80 °C until further analysis. Before measurement, MeOH was evaporated using a stream of nitrogen at room temperature and oxylipins were reconstituted in 150 µL reconstitution buffer (H_2O/acetonitrile/MeOH + 0.2% FA - 65:31.5:3.5), containing another set of 10-100 nM internal standards (5S-HETE-d8, 14.15-DiHETrE-d11, 8-iso-PGF2a-d4; Cayman Chemical). The prepared samples were separated using a Thermo Scientific Vanquish (UHPLC) system and a Kinetex C18 reversed phase column in the dimensions 150 * 2.1 mm and particle size of 2.6 µm (Phenomenex). The separation method comprised two eluent mixtures (A: water + 0.2% formic acid, B: 89.8% acetonitrile + 10% methanol + 0.2% formic acid) and started at 35% B, which was first kept constant for 1 min, then steadily increased to 90% B within 9 min and then further increased to 99% B within 0.5 min. 99% B was then kept constant for 5 min, before going back to 35% B within 0.5 min, which was kept constant for 4 min, resulting in a total run time of 20 min. Flow rate was kept at 200 µL min^{-1}, 20µL injection volume was used and column oven temperature was set to 40°C. For MS analysis, a Q Exactive HF Quadrupole-Orbitrap mass spectrometer (Thermo Fisher Scientific) was employed, comprising a HESI source which was run in negative ionization mode. Mass spectra were recorded operating from 250 to 700 m/z at a resolution of 60,000 at 200 m/z on MS1 level. The two most abundant precursor ions were selected for fragmentation (HCD 24 normalized collision energy) at a resolution of 15000 @ 200 m/z, preferentially ions from an inclusion list which contained 31 m/z values specific for oxylipins (Supplementary Table S2). For negative ionization, a spray voltage of 2.2 kV and a capillary temperature of 253 °C were applied, with the sheath gas set to 46 and the auxiliary gas to 10 arbitrary units. Raw files were analysed manually using Xcalibur Qual Browser (version 4.1.31.9; Thermo Fisher Scientific), using reference spectra from the Lipid Maps depository library from July 2018 [1]. For relative quantification the software TraceFinder (version 4.1; Thermo Fisher Scientific) was used.

Proteins

500 µL of sample were diluted in 2.5 mL absolute ethanol (AustroAlco) in 15 mL tubes (Falcon) and stored over night at -20 °C. Samples were then centrifuged for 30 min at 4536 g and 4 °C. The obtained protein pellets were used for an enzymatic digest using a modified filter-aided sample preparation (FASP) protocol as previously described [2]. Briefly, 20 µg of protein were loaded onto a 10 kD molecular weight cut-off filter (Merck). After reduction with dithiothreitol (Gerbu) and alkylation with iodoacetamide (Sigma-Aldrich) the protein digestion was achieved by applying Trypsin/Lys-C Mix (MS grade; Promega) twice for 16 and 4 h, respectively. The eluted peptide solution was dried via vacuum centrifugation and stored at -20 °C until further analysis. Upon analysis, dried peptide samples were thawed and redissolved in 5 µL 30% formic acid, supplemented with four synthetic peptide standards for internal quality control and further diluted with 40 µL of mobile phase A (97.9% H_2O, 2% acetonitrile, 0.1% formic acid; all VWR). 1 µL was injected into the Dionex UltiMate™ 3000 RSLC nano LC system coupled to a Q Exactive™ Orbitrap™ MS (all Thermo Fisher Scientific). Peptides were trapped on a C18 2 cm × 100 µm precolumn and LC separation was performed on a 50 cm × 75 µm Pepmap100 analytical column (Thermo Fisher Scientific). A gradient from 7% to 40% mobile phase B (79.9% acetonitrile, 20% H_2O, 0.1% formic acid) at a flow rate of 300 nL min^{-1} within 43 min was applied, leading to a total run time of 85 min including a washing and reequilibration step. Mass resolution on the MS1 level was set to 70000 (at m/z = 200) with a scan range from 400 to 1400 m/z. The top eight abundant peptide ions were chosen for fragmentation at 30% normalized collision energy, and resulting fragments were analyzed in the Orbitrap at a resolution of 17500 (at m/z = 200). For the identification and label free quantification of proteins MaxQuant software package (version 1.6.1.0) was used as previously described [3]. The human

UniProt database (version 03/2018, restricted to reviewed entries only) with 20316 entries was used for the search, and the false discovery rate (FDR) was set to 0.01 on both peptide and protein level. The alignment time window was set to 1 min, with a match time window of 5 min.

Amino acids

For amino acid analysis, 100 µL CSF were mixed with 100 µL 5% sulfosalicylic acid (Sigma Aldrich). After shaking the mixtures vigorously for 15 min at 8 °C on a thermoshaker, the solutions were centrifuged for 8 min at 4700 g at 10 °C. 25 µL of the supernatants were transferred into HPLC-vials, diluted with 145 µL acetonitrile (VWR) and 30 µL internal standard (^{13}C, ^{15}N labelled amino acid mix, Sigma Aldrich). Amino Acids were separated on a XBridge BEH Amide column (2.1 mm × 150 mm, 2.5 µm, Waters) with a XBridge Glycan BEH Amid pre-column (130 A. 2.5 µm, 2.1 mm × 5 mm, Waters) using a 1260 Infinity HPLC (Agilent Technologies) coupled to a 6420 TripleQuad LC/MS (Agilent Technologies). A gradient program was used starting at 94 % phase A (acetonitrile + 0.2 % formic acid) and 4 % phase B (water + 10 mM ammonium formate + 0.2 % formic acid) constantly increasing from minute 6 to 24 to 60 % phase A and 40 % phase B. After holding this condition for 4 min, the system was reequilibrated for 7 min. For detection of all analytes, two transitions in the positive ionization mode were used as mentioned in Supplementary Table S3. Gas temperature was set at 350 °C, gas flow at 12 L min^{-1}, nebulizer at 30 psi and capillary voltage at 4000 V. Resulting peak areas were normalized against the matching internal standard.

References

1. O'Donnell, V.B.; et al., LIPID MAPS: Serving the next generation of lipid researchers with tools, resources, data, and training. *Sci. Signal* **2019**, *12*(563).
2. Bileck, A.; et al., Comprehensive assessment of proteins regulated by dexamethasone reveals novel effects in primary human peripheral blood mononuclear cells. *J. Proteome Res.* **2014**, *13*(12), p. 5989–6000.
3. Neuditschko, B.; et al., The challenge of classifying metastatic cell properties by molecular profiling exemplified with cutaneous melanoma cells and their cerebral metastasis from patient derived mouse xenografts. *Mol. Cell Proteom.* **2019**.
